# Association between surgical volumes and real-world healthcare cost when using a mesh capturing device for pelvic organ prolapse: A 5-years comparison between single- versus multicenter use

**DOI:** 10.1007/s00192-021-04698-x

**Published:** 2021-02-26

**Authors:** Edward Morcos, Christian Falconer, Emilie Toresson Grip, Kirk Geale, Katarina Hellgren, Georgios Poutakidis, Daniel Altman

**Affiliations:** 1 Department of Clinical Sciences, Division of Obstetrics and Gynecology, Karolinska Institutet Danderyd Hospital, SE-171 77 Stockholm, Sweden; 2 Department of Gynecology & Obstetrics, Karolinska Institutet, Danderyd University Hospital, Danderyd, 182 88 Stockholm, Sweden; 3 Quantify Research, Stockholm, Sweden; 4 Department of Public Health and Clinical Medicine, Umeå University, Umeå, Sweden; 5 Department of Women’s and Children’s Health, Uppsala University, Uppsala, Sweden; 6 Stockholm Urogynecological Clinic, Stockholm, Sweden

**Keywords:** Health-care costs, Surgery volume, Vaginal prolapse mesh, Centralization

## Abstract

**Introduction and hypothesis:**

The aim of this study was to evaluate whether high surgical volume at a single center was associated with lower healthcare costs compared to lower surgical volume in a multicenter setting.

**Methods:**

All patients had symptomatic and anatomical apical prolapse (POP-Q ≥ stage II) with or without cystocele and were operated on by a standard surgical procedure using the Uphold mesh. Data on time of resource use in terms of surgery time, hospital stay and re-interventions across 5 years were compared between the single center (97 patients) and multicenter (173 patients, at 24 clinics). Unit costs for surgical time, inpatient and outpatient visits were extracted from the single-center hospital’s operation analysis program and prime production cost. Total costs were estimated for primary surgery and during 5-year follow-up.

**Results:**

Costs for primary surgery were comparable between the single and the multicenter ($13,561 ± 2688 and $13,867 ± 1177, *P* = 0.29). Follow-up costs 5 years after primary surgery were 2.8 times higher at the multicenter than single center ($3262 vs. $1149, *P* < 0.001). Mean cost per patient over 5 years was significantly lower at the single than multicenter [$14,710 (CI: 14,168–15,252) vs. $17,128 (CI: 16,952–17,305), *P* < 0.001)].

**Conclusions:**

Using a mesh kit for apical pelvic organ prolapse in a high surgical volume center was associated with reduced healthcare costs compared with a lower volume multiple-site setting. The cost reduction at the high surgical volume center increased over time because of lower surgical and medical re-intervention rates for postoperative complications and recurrence.

## Introduction

Long-term assessments of reconstructive surgery using the transvaginal Uphold^Lite^ mesh kit to suspend the apical vaginal segment have shown the procedure to improve pelvic organ prolapse symptoms and have high effectiveness in restoring the vaginal anatomy [1–4]. Although the Uphold^Lite^ mesh kit is no longer commercially available, ongoing research on the use of biomaterial implants is encouraged to continue by regulatory authorities in order to collect long-term safety and efficacy data. Accumulating evidence suggests that use of vaginal mesh is efficacious with relatively low rates of complications when performed at high surgical volume centers [1–8]. High surgical volumes lower the perioperative complication rates and re-operation rates compared to sites with low surgical volumes [5–8].

As healthcare expenditures are rising globally, well-informed decisions on allocation of scarce resources to optimize provision of healthcare, quality of treatment and patient safety are increasingly important [9]. Given that costs of inpatient care alone constitute approximately 25% of all health expenditures in the Nordic countries (https://www.oecd-ilibrary.org/social-issues-migration-health/health-at-a-glance-2019_4dd50c09-en), improving surgical cost-effectiveness is of particular relevance. Centralization of surgical procedures includes concentration of resources, including staff, material and knowledge, allowing for high surgical volumes at a single site. Advocates of surgical centralization suggest that in a particular field the concentration of resources to high-volume centers may lead to improved quality of care and potentially increased economic efficiency [10–13]. The aim of this study was to investigate the difference in healthcare costs between a high surgical volume single site and low surgical volume multicenter sites using a mesh capturing device in a standardized surgical procedure.

## Materials and methods

### Patients and surgery centers

Data from two previously published cohort studies comparing outcomes from high surgical volume at a single-center to low surgical volumes in a multicenter setting was used [1, 3, 6, 14]. At the single center, 115 patients were operated on by two experienced urogynecological surgeons from January 2012–December 2014 at the Department of Obstetrics and Gynecology, Danderyd Hospital, Stockholm, Sweden. Two hundred seven patients were operated on during 2012 at the multicenter setting by 26 surgeons at 24 centers spread across four countries: Sweden (11 centers), Finland (4 centers), Denmark (4 centers) and Norway (5 centers) [14]. All surgeons at the single- and multicenter sites were at a senior consultant level and obtained standardized pre-trial hands-on training. Surgery data, outcomes and re-interventions for the patient cohorts over 5 years were previously published in detail [1, 3, 6, 14]. Ninety-seven patients operated on by two surgeons (E.M. 57 and C.F. 40) were available for the cost evaluation at the single center and were compared to 173 patients operated on by 26 surgeons (range 1–13 per surgeon) at the multicenter.

All patients had symptomatic and quantified apical segment prolapse (POP-Q ≥ stage 2) with or without anterior wall prolapse according to the pelvic organ prolapse quantification (POP-Q) system [15]. Using a standardized surgical procedure with a capturing device, the Uphold transvaginal mesh was placed to suspend the apical vaginal segment [6, 14]. POP-Q stage 0 or 1 in the apical compartment was considered an optimal anatomical outcome and was the primary outcome measure for the analysis.

As previously reported, the study protocol was almost identical for both studies, and neither study was blinded [6, 14]. Patients with current or previously treated pelvic organ cancer and cervical elongation were excluded, as were those with severe rheumatic disease, insulin-treated diabetes mellitus, connective tissue disorders, current systemic steroid treatment or urinary incontinence. Patient follow-up occurred 1 and 5 years after surgery in the multicenter setting study and after 2 and 5 years in the single-center study. There were no restrictions on weight, parity, menopausal status or previous surgery in either studies. Index date was considered the date of primary surgery using the Uphold Mesh.

Resource use was estimated based on previously collected data on time of surgery, recovery care time, type of anesthesia, length of hospital stay, outpatient visits and prescription drug use. Medical resource use was extracted from previously collected data for primary surgery and for medical and surgical re-interventions over a 5-year period [1, 3, 6, 14]. Resource use for primary surgical complications including urinary tract infection and hematoma, etc., were based on required medical treatment at an out-clinic or hospital re-admission, e.g., for embolization of the uterine artery [6, 14].

### Cost estimation

Costs per surgical minute (30 USD), inpatient day (1,024 USD) and outpatient visit (362 USD) were estimated based on charges derived from the single-center internal analysis system fee-for-service schedules, prime production cost and real prices from negotiated agreements for equipment and disposables. For consistency, the same unit costs were used to estimate the total cost of resource use from all sites in all countries at the multicenter. Unit costs included surgeon and personal salaries, equipment and supplies, mesh kit, nursing services, prescriptions, room-related services, local rent, laboratory tests and radiological examination. Costs of pharmacy-dispensed medication were based on pharmacy retail prices in Sweden (https://www.fass.se/LIF/startpage) and used to estimate the costs of pharmacy-dispensed medications in all countries. Costs of hospital-administered drugs were based on costs provided by the single-center main pharmacy supplier and used for all countries.

Total healthcare costs for the comparative analysis between the single and the multicenter were calculated by multiplying the common unit costs with site-specific resource use for surgery and recovery time, hospital stays, outpatient visits and pharmacy-dispensed medications. Costs were estimated separately for primary surgery and for the follow-up time up to 5 years. Indirect costs capturing differences in productivity losses between patients treated in single or multicenter settings, for example, were not available for inclusion, nor were certain overheads.

To analyze whether the type of anesthesia was associated with surgery time and thus a potential confounder in the comparison between the single- and multicenter sites, operation time data from the single center were analyzed and divided into two groups based on the type of anesthesia: spinal or general anesthesia. Time (minutes) at the operation ward included the operating room time and time at the recovery unit after surgery until the patient was discharged from the operation ward to an in-patient ward (hospital stay). Operating room time was divided into preparation time before anesthesia start, interventional anesthetic time before surgery, surgery time and anesthetic time after surgery. Time spent at the recovery unit was considered as the time from patient discharge from the operation room to the recovery unit until patient discharge to the in-patient department (hospital stay).

### Statistical analyses

IBM^@^SPSS^©^ Statistics (version 25, Chicago, IL, USA, 2017) was used for all statistical analyses performed. Mean differences in costs and resource use between the single and the multicenter were evaluated using independent sample two-sided t-tests. Chi-squared test of independence was used to test the statistical difference between nominal data. Repeated measures ANOVA was used to evaluate differences over time within groups and to compare between groups. *P*-values < 0.05 were considered statistically significant. We used multiple regression stratified analysis of covariance (ANCOVA) to investigate whether patient’s socioeconomic, medical and surgery characteristics were predictive of costs. Stratified analysis was also performed to evaluate whether the impact of regressors on costs differs between the sites, using standardized beta-coefficient values. Observations with missing information were dropped, and there was no imputation of missing data. Number of observations is shown for all results.

## Results

### Demographics

Patients medical, socioeconomic and surgery characteristics are illustrated in detail in Table 1. There was a significant difference at the education level to the favor of the single center (*P* < 0.001). Use of spinal anesthesia was more common at the single center [82/97 (84.5%) vs. 87/173 (50.3%), *P* < 0.001], whereas general anesthesia was more commonly used at the multicenter [86/173 (49.7%) vs. 15/97 (15.5), *P* < 0.001]. Operating time was longer at the single center (63.5 ± 14.3 vs. 56.5 ± 18.4, *P* = 0.001) probably because of the higher rate of previous prolapse recurrence surgery [57/97 (58.8%) vs. 66/173 (38.2%), *P* = 0.001]. Hospital stay was shorter at the single center (1.3 ± 0.63 vs. 1.7 ± 0.96, *P* < 0.001).

**Table 1:** Demographic, medical, socioeconomic and surgery characteristics for the single and multicenter settings

	Single center		Multicenter		*P*-value
	Mean ± SD	*n*	Mean ± SD	*n*	
Age (≤ 65, > 65 year)	57.7 ± 7.6 (45.4%), 73 ± 4.3 (54.6%)	97	58.2 ± 6.8 (43.8%), 72.6 ± 5.3 (56.2%)	169	0.804
BMI	25.7 ± 3.6	81	25.8 ± 3.01	164	0.859
Vaginal deliveries	2.4 ± 1.3	86	2.2 ± 1.06	166	0.160
Somatic diseases (none, CVS, other diseases)	34 (35.1%), 39 (40.2%), 24 (24.7%)	86	60 (34.7%), 75 (43.4%), 38 (22%)	173	0.837
Physical training (0, 1–2, 3–4 times/week)	8 (9.8%), 45 (54.9%), 29 (35.4%)	82	16 (9.9%), 96 (59.6%), 49 (30.4%)	161	0.732
Job (in pension, working)	57 (67.1%), 28 (32.9%)	85	91 (60.7%), 59 (39.3%)	150	0.330
Education level (elementary, upper secondary school, university education 3–6 years)	23 (27.1%), 18 (21.2%), 44 (51.7%)	85	58 (34.9%), 64 (38.6%), 44 (26.5%)	166	< 0.001
Annual income (< $29,000, > $29,000/year)	38 (50.7%), 37 (49.3%)	75	64 (41.3%), (58.7%)	155	0.180
Pain (VAS 0–10)	1.1 ± 1.85	88	1.0 ± 1.61	165	0.661
Hysterectomy prior to surgery vs. still having uterus	17 (17.5%), 80 (82.5%)	97	69 (39.9%), 104 (60.1%)	173	< 0.001
Primary vs. recurrent prolapse surgery	40 (41.2%), 57 (58.8%)	97	107 (61.8%), 66 (38.2%)	173	0.001
Anesthesia type (spinal, general)	82 (84.5%), 15 (15.5%)	97	87 (50.3%), 86 (49.7%)	173	< 0.001
Operating time (min)	63.5 ± 14.3	97	56.5 ± 18.4	173	0.001
Hospital stay (mean ± SD) (≤ 1, > 1 day)	1.3 ± 0.63 75 (77.3%), 22 (22.7%)	97	1.7 ± 0.96 70 (42.2%), 96 (57.8%)	166	< 0.001 < 0.001

Independent sample two-sided t-test and chi-squared test of independence were used. *P* < 0.05 was considered significant

### Assessing diverging anesthesia routines in single and multicenter sites

Operation and recovery time by type of anesthesia (spinal and general) at the single center is presented in Table 2. The total estimated time spent in the operation department, from start of operating theater time to patient discharge from the postoperative recovery unit, was comparable between the spinal and general anesthesia patients, respectively [351 ± 82.7 (CI: 333–369) vs. 353 ± 96.8 (CI: 300–407) min, *P* = 0.922]. The overall time at the operation department (operation theater and recovery unit) was comparable between the single and the multicenter [351.2 ± 84.5 (CI: 334.1–368.2) vs. 343 ± 18.6 (CI: 340.2–345.8) min, *P* = 0.351]. Furthermore, no significant differences in the overall time at the operation department were detected when comparing spinal (350.8 ± 82.7 vs. 339.4 ± 18.4, *P* = 0.224) to general anesthesia (353.1 ± 96.8 vs. 346.7 ± 18.2, *P* = 0.802) for the single and the multicenter, respectively, indicating that differences in mode of anesthesia do not appear to drive differences in resource use related to surgery time when comparing low-volume sites with the high-volume site. The only significant observation detected was the comparison of overall time at the operation department between spinal and general anesthesia at the multicenter (339.4 ± 18.4 vs. 346.7 ± 18.2, *P* = 0.009).

**Table 2:** Time data extracted for the primary surgery (minutes)

	Spinal anesthesia	General anesthesia	Full population (spinal and general)	*P*-value (spinal vs. general)
	*n* = 82	*n* = 15	*n* = 97	
Operation theater time before anesthesia	13 ± 8 (CI: 11–15)	12 ± 5 (CI: 9–15)	13 ± 7 (CI: 11–14)	0.674
Interventional anesthesia time before surgery	14 ± 6 (CI: 13–15)	12 ± 6 (CI: 9–16)	14 ± 6 (CI: 13–15)	0.328
Surgery time	63 ± 14 (CI: 60–66)	68 ± 16 (CI: 59–77)	64 ± 14 (CI: 61–66)	0.210
Interventional anesthesia time after surgery	14 ± 5 (CI: 13–15)	18 ± 6 (CI: 14–21)	15 ± 5 (CI: 14–16)	0.021
Total operation theater time	104 ± 23 (CI: 100–108)	110 ± 23 (CI: 97–123)	105 ± 19 (CI: 101–109)	0.283
Postoperative recovery care time	223 ± 81 (CI: 205–241)	216 ± 95 (CI: 165–269)	222 ± 82 (CI: 205–238)	0.771
Mean time without surgery time	288 ± 82 (CI: 270–307)	285 ± 97 (CI: 232–339)	287 ± (CI: 278–333)	0.908
^†^Total time at operation department	351 ± 82.7 (CI: 333–369)	353 ± 96.8 (CI: 300–407)	351 ± 84.5 (CI: 334–368)	0.922

Data were extracted from the single-center hospital’s operation analysis program and are shown as mean ± SD and CI

^**†**^Total time at the operation department is calculated from patient start time at the operation theater to departure from the recovery care unit. Independent sample two-sided t-test, *P* < 0.05 was considered significant

### Primary surgery cost comparison

Disaggregated total costs for primary surgery at the single and multicenter using the Uphold mesh are shown in Table 3. Lower cost for surgery time was estimated at the multicenter compared to the single center ($1704 ± 554 vs. $1914 ± 432, *P* < 0.001). Costs for interventional anesthesia before and after surgery, including equipment and drugs used, were lower at the single center ($465 ± 200 vs. $587 ± 166 and $444 ± 152 vs. $482 ± 60, *P* < 0.001 and 0.019, respectively). Despite these differences, there was no significant difference between centers when the total operation theater ($3207 ± 610 vs. $3165 ± 623, *P* = 0.591) and recovery unit costs ($6684 ± 2487 vs. $6,557 ± 76, *P* = 0.614) were compared. Cost for hospital stay was higher at the multicenter than single center ($2481 ± 985 vs. $2043 ± 644, *P* < 0.001). Total costs for the primary surgery were comparable between the single and multicenter ($13,561 ± 2688 vs. $13,867 ± 1177, respectively, *P* = 0.288).

**Table 3:** Costs for primary surgery with the Uphold mesh

	Single center			Multicenter			*P*-value (cost differences)
	Mean ± SD	CI	*n*	Mean ± SD	CI	*n*	
Operation costs							
Surgery time	1914 ± 432	1827–2002	97	1704 ± 554	1621–1787	173	0.001
Total surgery cost (including mesh)	2796 ± 432	2709–2883	97	2586 ± 554	2503–2697	173	0.001
Anesthesia before surgery start^**†**^	465 ± 200	425–505	97	587 ± 166	562–612	173	< 0.001
Anesthesia after surgery ended^**††**^	444 ± 152	413–474	97	482 ± 60	473–491	173	0.019
Total operation theater cost	3207 ± 610	3084–3330	97	3165 ± 623	3071–3258	173	0.591
Recovery unit cost	6684 ± 2487	6183–7185	97	6557 ± 76	6545–6568	173	0.614
Total cost at operation department	10,636 ± 2450	10,122–11,150	97	10,505 ± 616	10,412–10,597	173	0.618
Cost of hospital stay	2043 ± 644	1913–2172	97	2481 ± 985	2333–2629	173	< 0.001
Total cost for spinal anesthesia	13,503 ± 2648	12,921–14,084	82	13,529 ± 699	13,380–13,678	87	0.930
Total cost for general anesthesia	13,878 ± 2975	12,230–15,525	15	14,209 ± 1440	13,900–14,518	86	0.678
Total costs of primary surgery^†††^	13,561 ± 2688	13,019–14,102	97	13,867 ± 1177	13,691–14,187	173	0.288

Costs are shown as mean ± SD and CI, in USD ($)

^**†**^ and ^**††**^Other costs for anesthesia including equipment and pharmacy are included

^**†††**^Total costs for primary surgery include costs for hospital stay and two standard outpatient visits

Analysis was done by independent sample two-sided t-test; *P* < 0.05 was considered significant

### Follow-up cost comparison

**Fig. 1:**
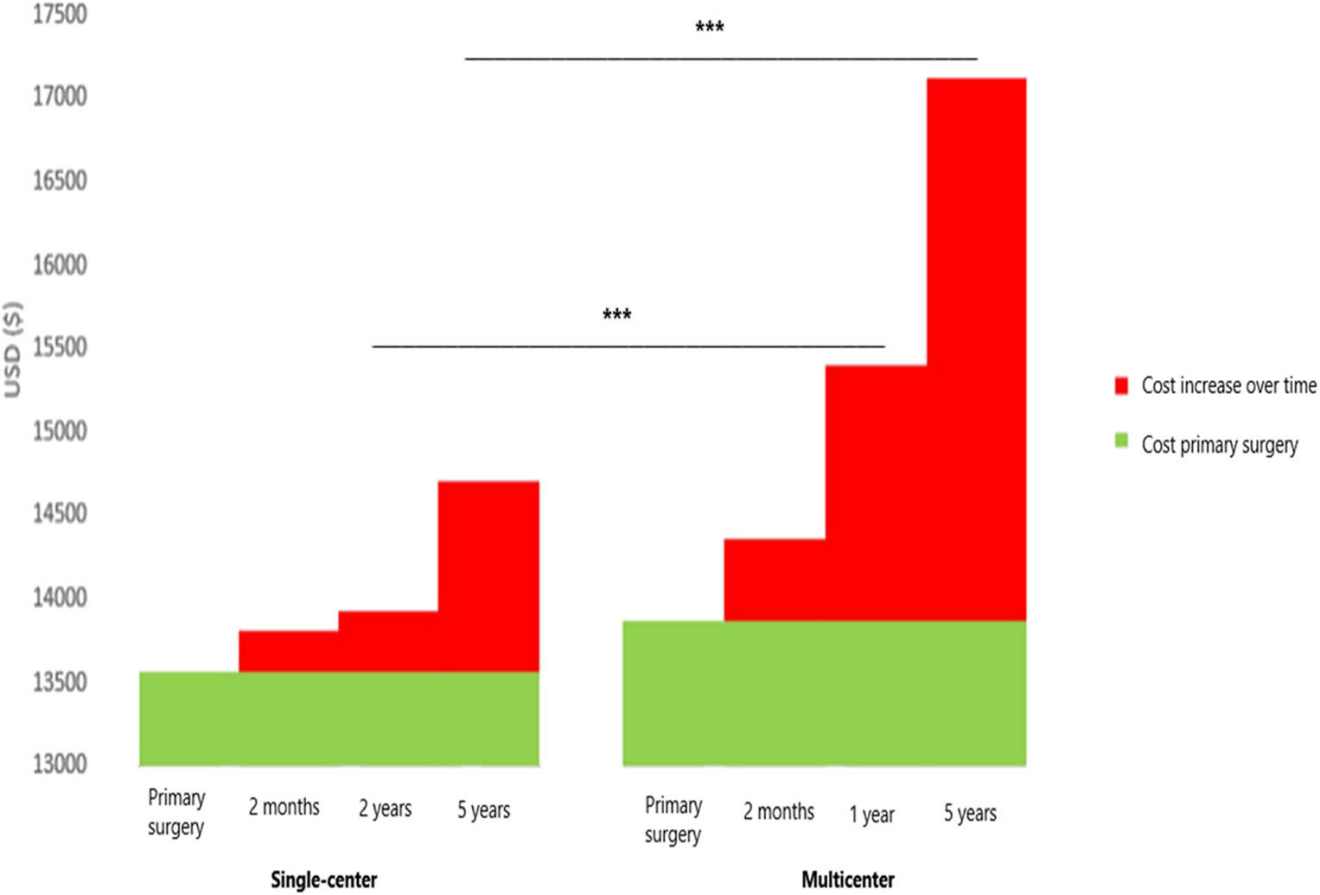
Mean cost per patient for 5 years including costs for primary surgery with the Uphold mesh (green staple) and follow-ups costs over 5 years after surgery (red staples). Costs are shown as mean with confidence interval (CI). ***P < 0.001, ANOVA repeated measures

Follow-up costs for direct medical and surgical re-interventions after primary surgery and up to 2 months were doubled in the multicenter compared to single center use (mean = $497 vs. $244, *P* =  < 0.001) (Table 4 and Fig. 1). Costs included medical and surgical intervention for direct and indirect surgery complications and mesh-related complications in a total of 21 patients at the multicenter including urinary tract infection *n* = *2*, fever *n* = *1*, vaginal hematoma *n* = 2, groin pain *n* = 7, inferior pudendal artery embolization *n* = *1* and reoperation and other intervention for mesh complications *n* = *5* to be compared to six patients at the single center including urinary tract infection *n* = *3*, wound infection *n* = *1* and reoperation for secondary bleeding *n* = *2*. Total costs estimated from primary surgery and up to 2 months after surgery were $14,364 (CI: 14,187–14,540) vs. $13,805 (CI: 13,263–14,363) for the multicenter and the single center, respectively (*P* = 0.054).

**Table 4:** Total costs ($) including accumulating follow-up costs at the single center (*n* = 97) and multicenter (*n* = 173)

	Single center		Multicenter		*P*-value (total cost differences)
	Total cost mean (CI)	Follow-up cost	Total cost mean (CI)	Follow-up cost	
Primary surgery	13,561 (13,019–14,102)		13,867 (13,691–14,187)		0.288
2 months	13,805 (13,263–14,347)	244	14,364 (14,187–14,540)	497	0.054
1–2 years	13,933 (13,391–14,475)	128	15,403 (15,226–15,579)	1039	< 0.001
5 years	14,710 (14,168–15,252)	777	17,128 (16,952–17,305)	1726	< 0.001

*P* < 0.05 was considered significant, repeated measures ANOVA

The estimated follow-up costs from 2 months to the next follow-ups (1 year for multicenter, 2 years for single center) were $1039 vs. $128 (*P* < 0.001) for the multicenter vs. the single center, leading to a significant increase of total costs at the multicenter [$15,403 (CI: 15,226–15,579) vs. $13,933 (CI: 13,391–14,475), *P* < 001]. The follow-up cost corresponded to reoperation for prolapse recurrence, *n* = *7*, and mesh revisions, *n* = *4*, at the multicenter compared to mesh revision, *n* = *1*, at the single center.

At 5 year follow-up, there was further increase of follow-up costs by $1726 at the multicenter compared to $777 at the single center (*P* < 0.001). Additional follow-up costs at 5 years were because of reoperation for prolapse recurrence, *n* = *16* patients at the multicenter and *n* = *6* patients at the single center, and hysterectomy because of pain at the multicenter, *n* = *1* patient. The total follow-up cost across 5 years was $3262 at the multicenter compared to $1149 at the single center (*P* < 0.001). In detail, mean follow-up cost for medical and surgery interventions including out-clinic and readmission because of surgery complications and mesh related complications was $1001 at the multicenter vs. $372 at the single center (*P* < 0.001). For reoperation of prolapse recurrence, the mean follow-up cost was $2260 at the multicenter vs. $777 at the single center (*P* < 0.001). Thus, the mean total cost per patient through 5 years was significantly higher at the multicenter compared to the single center [$17,128 (CI: 16,952–17,305) vs. $14,710 (CI: 14,168–15,252), *P* < 0.001] (Table 4 and Fig. 1).

### Effects of patient medical, socioeconomic and surgery characteristics on costs

All results of multiple regression analysis on the impact of patient medical, socioeconomic and surgical characteristics on costs by type of site are shown in Table 5. Of patient medical and socioeconomic characteristics, only higher educational level at the single center (β -coefficient -1307, CI: $ -247– -143 and *P* = 0.029) inversely correlated to primary surgery and total costs at 5 years compared to the multicenter (difference in standardized β-coefficient -1.562).

**Table 5:** Multiple regression and stratified analysis of covariates (patient’s medical, socioeconomic and surgery characteristics) for primary surgery and total costs at 5 years

	Single center				Multicenter				Difference in standardized β-coefficient
	β-coefficient	CI	*P-value*	*n*	β-coefficient	CI	*P-value*	*n*	
Age (≤ 65 vs. > 65 year)	−141.5	−2111– 1828	0.886	97	−84.8	−534.4– 364.8	0.709	169	0.117
BMI (< 25 vs. 25–29.9 vs. ≥ 30)	−118.9	−359– 121	0.324	81	9.06	−53.2– 71.3	0.773	164	−0.641
Vaginal deliveries	−13.7	−703– 675	0.968	86	5.88	−157.0– 168.8	0.943	166	−0.056
Somatic diseases (CVS vs. other diseases)	−361.3	−1499– 776	0.526	97	−40.2	−297.9– 217.5	0.757	173	−0.162
Physical training (0 vs. 1–2 vs. 3–4/week)	−916.3	−2209– 377	0.161	82	−41.3	−341.3– 258.7	0.785	161	−0.571
Job (in pension vs. working)	−644	−3000– 1711	0.585	85	0.071	−482.2– 482.4	0.999	150	−0.571
Education level (elementary vs. upper secondary school vs. university education 1–3 vs. 4–6 years)	−1307	−2471– -143	0.029*	85	108	−137.6– 353	0.385	167	−1.562 *
Annual income (< $29,000 vs. > $29,000/year)	531.2	−1539– 2601	0.608	75	−172.3	−560.9– 216.4	0.381	155	0.700
Pain (VAS 0–10)	−14.9	−645– 675	0.964	88	20.7	−109.6– 151.1	0.753	165	−0.137
Hysterectomy prior to surgery vs. still having uterus	−64.5	−2116– 1987	0.95	97	−146.8	−543.8– 250.2	0.465	173	0.338
Primary vs. recurrent prolapse surgery	−489.5	−2131– 1152	0.551	97	−103.3	−488– 282	0.596	173	−0.030
Anesthesia type	−118.1	−2396– 2160	0.917	97	669.5	304– 1035	< 0.001*	173	−1.883 *
(Spinal vs. general)									
Operating time	48.2	−21.4– 117.7	0.170	97	38.5	28.2– 48.8	< 0.001*	173	−3.042 *
(min)									
Hospital stay	2205	−301– 4309	0.025*	97	1594	1212–1976	< 0.001*	166	−3.025 *
(≤ 1 vs. > 1 day)									

Results are shown as β-coefficient, confidence intervals (CI) and *P*-values. *P* < 0.05 was considered significant

Differences in standardized β-coefficient values of < –1 or >  + 1 between the single and the multicenter were considered significant

^*^ Indicate statistical significance or not

From the surgical characteristics, more common use of general anesthesia in the multicenter setting was associated with an increase of costs of primary surgery and total costs at 5 years (β-coefficient 669.5 CI: $304–1035, *P* < 0.001; difference in standardized β-coefficient of -1.883). Also, longer hospital stay in the multicenter setting was associated with an increase of costs of primary surgery and total costs at 5 years (β-coefficient 1593.9, CI: $1212–1976, *P* < 0.001 and difference in standardized β-coefficient of -3.025). However, shorter operation times in the multicenter setting correlated to lower primary surgery costs and total costs at 5 years (β-coefficient 38.5, CI: $28.2–$48.8, *P* < 0.001; difference in standardized β-coefficient -3.042). No other characteristics had a significant influence on costs.

## Discussion

This long-term assessment compared real-world healthcare costs between two separate cohort studies conducted in a high-volume single-center setting with multicenter low surgical volume using the same prolapse mesh kit in a standard surgical procedure. We found that costs for primary surgery were comparable between the single and multicenter and were comparable to previously estimated costs for vaginal surgery [16]. However, the long-term follow-up costs were more than two times higher in the multicenter setting, and single-site high-volume use reduced healthcare utilization costs in the long term. Surgery at a high-volume single center was associated with 65% lower follow-up costs and 14% lower total costs after 5 years compared to average costs at multicenter low-volume sites. This corresponds well with a previous study comparing the two cohorts that showed lower complication rates, re-intervention rates, lower risk for prolapse recurrence and significantly improved anatomical outcomes at the single compared to multicenter 5 years after primary surgery [3].

The difference in costs may partially be explained by more postoperative complications in the multicenter setting. The largest cost difference was found at 2 years after surgery where costs were eight times higher at the multicenter setting compared to the single center. This was mainly attributed to costs associated with re-operations for prolapse recurrence. At the end of follow-up at year 5, the cost difference in follow-up costs had decreased but remained significantly different and more than twice as large at the multicenter compared to the single center. Total costs 5 years after surgery were also significantly lower at the high-volume single site compared to the multicenter sites.

The impact of centralization of healthcare activities on healthcare costs has been the topic of many recent studies. Centralization and reliance on newer surgical techniques of total hip replacement (THR) services have been shown to reduce costs [10]. Undergoing radical cystectomy at a high-volume medical center was associated with improved outcomes and reduced costs [12]. High-volume hospitals have also been shown to provide more cost-effective neurosurgical care, and centralization of care at high-volume neurosurgical institutions may be a promising strategy for delivering higher value care, achieving better outcomes at lower costs [13]. Current treatment of ovarian cancer patients in semi-specialized hospital settings is a cost-effective strategy [17]. Despite the mounting evidence that centralization of surgical procedures increases efficiency and cost-effectiveness, the evidence to support this change in practice is scarce and understudied in advanced pelvic organ prolapse surgery.

Accumulating evidence suggests that the Uphold vaginal mesh may be effective and safe if surgery is done at a high surgery volume unit [1–7]. In the present study, potential causes of cost reduction at the single high-volume center were lower complication rates, lower rates of prolapse recurrence and fewer surgical and medical re-interventions. Other factors that may have increased the difference in costs despite the standardization of the procedure were the longer postoperative hospital stay and more frequent use of general anesthesia at the multicenter setting.

In search of predictors that may have influenced costs, we performed a multiple regression and stratified analysis of covariances. Only a higher educational level at the single center predicted lower costs of primary surgery as well as total costs at 5 years. In previous studies, the educational level had an impact on patient-reported disease-specific pelvic floor outcomes and was related to improved health-related quality of life compared to lower educational levels [18, 19]. It is not known exactly how educational level affects costs, but one may assume that a higher educational level is associated with an overall healthier lifestyle, less obesity, less tobacco use and fewer comorbidities. Surgical characteristics including type of anesthesia, operating time and length of hospital stay independently influenced costs. Routine use of spinal anesthesia lowered the costs in favor of the high-volume center, whereas longer operating time, probably explained by more patients with prolapse recurrence at the time of primary mesh surgery, was an economic disadvantage at the single high-volume center.

Strengths of the study are use of real-world data on time and costs recorded at the hospitals, capturing resource use in clinical practice. The study populations were sufficiently large to determine the impact of the most important surgical factors on the costs associated with the procedure. Furthermore, classification of the procedure was homogeneous since all operations used an identical mesh kit and were performed in a standardized surgical manner.

Patient and surgeon allocation to high- and low-volume sites was not randomized, suggesting that several unmeasured effects may to some extent constitute a source of bias in estimates of costs associated to each setting. Another limitation was that the number of patients was insufficient for an analysis on the importance of individual experience and skill of the surgeons involved. Also, we did not have access to detailed socioeconomic characteristics of the patients to allow for an analysis of the influence of patient-attributed factors other than educational level. Since it has been argued that centralization should not be based solely on a minimum number of procedures, but rather on the multidisciplinary treatment of complex diseases [11], future studies should aim to control for these factors when comparing centralized single units to multicenter settings to fully understand the optimal setting for conducting mesh surgical procedures for pelvic organ prolapse. Additional ongoing studies are also warranted to compare the cost-effectiveness of vaginal mesh surgery to laparoscopic and robotic-assisted sacral colpopexy [20, 21] for apical pelvic organ prolapse.

## Conclusion

In this long-term multivariate cost analysis study, we found that using a mesh kit for apical pelvic organ prolapse in a high surgical volume center was associated with reduced healthcare costs compared to a lower volume multiple-site setting. The cost reduction at the high surgical volume center increased over time because of the lower surgical and medical re-intervention rates for postoperative complication and recurrence. These results may guide future regulatory and health economic decisions when planning and implementing new healthcare routines in urogynecological surgery.
